# Identify the immune characteristics and immunotherapy value of CD93 in the pan-cancer based on the public data sets

**DOI:** 10.3389/fimmu.2022.907182

**Published:** 2022-10-27

**Authors:** Aiyuan Guo, Jingwei Zhang, Yuqiu Tian, Yun Peng, Peng Luo, Jian Zhang, Zaoqu Liu, Wantao Wu, Hao Zhang, Quan Cheng

**Affiliations:** ^1^ Department of Dermatology, The Third Xiangya Hospital, Central South University, Changsha, China; ^2^ Department of Neurosurgery, Xiangya Hospital, Central South University, Changsha, China; ^3^ National Clinical Research Center for Geriatric Disorders, Xiangya Hospital, Central South University, Changsha, China; ^4^ Department of Infectious Disease, Zhuzhou Central Hospital, Zhuzhou, China; ^5^ Teaching and Research Section of Clinical Nursing, Xiangya Hospital of Central South University, Changsha, China; ^6^ Department of Oncology, Zhujiang Hospital, Southern Medical University, Guangzhou, China; ^7^ Department of Interventional Radiology, The First Affiliated Hospital of Zhengzhou University, Zhengzhou, China; ^8^ Department of Oncology, Xiangya Hospital, Central South University, Changsha, China; ^9^ Department of Neurosurgery, The Second Affiliated Hospital, Chongqing Medical University, Chongqing, China; ^10^ Clinical Diagnosis and Therapy Center for Glioma of Xiangya Hospital, Central South University, Changsha, China

**Keywords:** CD93, pan-cancer, immunotherapy, macrophages, T cells, tumor microenvironment

## Abstract

CD93 is a transmembrane receptor that is mainly expressed on endothelial cells. A recent study found that upregulated CD93 in tumor vessels is essential for tumor angiogenesis in several cancers. However, the underlying mechanisms are largely unexplored. Our present research systematically analyzed the characteristics of CD93 in tumor immunotherapy among 33 cancers. CD93 levels and co-expression of CD93 on cancer and stromal cells were detected using public databases and multiple immunofluorescence staining. The Kaplan-Meier (KM) analysis identified the predictive role of CD93 in these cancer types. The survival differences between CD93 mutants and WT, CNV groups, and methylation were also investigated. The immune landscape of CD93 in the tumor microenvironment was analyzed using the SangerBox, TIMER 2.0, and single-cell sequencing. The immunotherapy value of CD93 was predicted through public databases. CD93 mRNA and protein levels differed significantly between cancer samples and adjacent control tissues in multiply cancer types. CD93 mRNA expression associated with patient prognosis in many cancers. The correlation of CD93 levels with mutational status of other gene in these cancers was also analyzed. CD93 levels significantly positively related to three scores (immune, stromal, and extimate), immune infiltrates, immune checkpoints, and neoantigen expression.. Additionally, single-cell sequencing revealed that CD93 is predominantly co-expressed on tumor and stromal cells, such as endothelial cells, cancer-associated fibroblasts (CAFs), neutrophils, T cells, macrophages, M1 and M2 macrophages. Several immune-related signaling pathways were enriched based on CD93 expression, including immune cells activation and migration, focal adhesion, leukocyte transendothelial migration, oxidative phosphorylation, and complement. Multiple immunofluorescence staining displayed the relationship between CD93 expression and CD8, CD68, and CD163 in these cancers. Finally, the treatment response of CD93 in many immunotherapy cohorts and sensitive small molecules was predicted from the public datasets. CD93 expression is closely associated with clinical prognosis and immune infiltrates in a variety of tumors. Targeting CD93-related signaling pathways in the tumor microenvironment may be a novel therapeutic strategy for tumor immunotherapy.

## Introduction

Cancer remains the second most common cause of death in the United States and remains a significant global public health threat until now (1). Uncontrolled cell proliferation is mainly due to the accumulation of genetic and epigenetic alterations, leading to cancer formation (2, 3). Cancer occurs due to the synergistic action of multiple carcinogens, such as chemical and physical carcinogens, viruses, and bacteria (4, 5). There are only a few options for cancer treatment, including surgery, chemotherapy, targeted drug therapy, radiation and hormone therapy, stem cell transplant, clinical trials, and immunotherapy (6–8). Despite rapid improvements in early diagnosis and treatment in past decades, five-year survival rates for many cancer types remain unsatisfied (9). The past decades have witnessed tremendous developments in cancer immunotherapy, one of the most promising fields for the future of cancer treatment. Immunotherapy functions by inducing the immune system to target the tumor and stroma cells *via* various xenoantigens, ultimately enhancing the innate anti-tumor immune responses (10, 11). In particular, checkpoint inhibitors in the tumor microenvironment (TME) have been shown improve prognosis for patients with advanced malignancies, including melanoma, lymphoma, lung and bladder cancers (12, 13). A large number of immune checkpoints have been discovered through public databases according to the rapid development of high-throughput sequencing technology (14–16). Validating the effectiveness of these immune checkpoints through preclinical and clinical studies will help significantly improve the prognosis of cancer patients.

Recent evidence indicated CD93 act as anew immune checkpoint for immunotherapy in the TME (17–19). CD93 is a transmembrane protein from the Group XIV C-Type lectin family (20). It contains a short cytoplasmic tail, a C-type lectin domain, a unique transmembrane like and a highly glycosylated mucin like as well as a series of epidermal growth factor like structural domains (21). CD93 plays a vital role in endothelial cell-cell adhesion, cell migration, cell polarization, and phagocytosis (22). Endothelial cell migration is essential for angiogenesis and promotes the formation of new blood vessels under physiological and pathological conditions (23, 24). In addition, CD93 can regulate β_1_ integrin activation and fibronectin fibril formation, thereby mediating angiogenesis during tumorigenesis and growth. CD93 is expressed by various cell types, such as myeloid lineage, platelets, monocytes, microglia, and endothelial cells (25). CD93 expression levels in tumor blood vessels are associated with poor survival in patients with high-grade astrocytic glioma (21). Similarly, CD93-deficient glioma mice had significantly slower intracranial tumor growth and improved survival than wild-type mice. In addition, recent papers have shown that CD93 is a prognostic marker for many malignant cancers and is involved in immune responses in the TME during cancer immunization (26, 27). However, the specific mechanisms of CD93 in tumor immunity remain largely undiscovered. Thus, a comprehensive assessment of the predictive value of CD93 in other cancers and the co-expression and role of CD93 on tumor and stromal cells in the TME require further elaboration.

Therefore, in this paper, we systematically checked the prognostic and immune role of CD93 in pan-cancer based on public databases like TCGA, CCLE, and GTEX. Meanwhile, the survival difference between CD93 mutant and WT, CNV groups, and methylation were explored. Moreover, the co-expression of CD93 on various cell types in the TME were verified through the online dataset, single-cell sequencing analysis, and multiple fluorescent staining. Furthermore, the immunotherapy effectiveness and sensitive drugs targeting CD93 in these cancers were predicted.

## Materials and methods

### Data collection and preparation

The transcriptomic data of CD93 in pan-cancer cohorts were obtained from The Cancer Genome Atlas (TCGA; http://cancergenome.nih.gov) (28) and Genotype Tissue-Expression (GTEX; https://gtexportal.org/home/) (29) databases. The cell lines data were collected from the Cancer Cell Line Encyclopedia (CCLE; https://sites.broadinstitute.org/ccle/) (30) dataset. The single-cell sequencing data were collected from the Gene Expression Omnibus (GEO; https://www.ncbi.nlm.nih.gov/geo/) database, including BLCA (GSE145137), BRCA (GSE75688 and GSE118389), CHOL (GSE125449), COAD (GSE81861), HNSC (GSE103322), KIRC (GSE121636 and GSE171306), LIHC (GSE125449), OV (GSE118828), PRAD (GSE137829), SKCM (GSE72056), and STAD (GSE183904). The Single Cell Portal platform was used to collect the scRNA-seq dataset of GBM (SCP50 and SCP393, http://singlecell.broadinstitute.org). The Genome Sequence Archive (GSA) database was used to collect the scRNA-seq dataset of PAAD (CRA001160, https://ngdc.cncb.ac.cn/gsa/browse/CRA001160). The BioProject (#PRJNA591860) database was applied to collect the scRNA-seq dataset of LUAD.

### CD93 prognostic and immune role identification

The Kaplan-Meier (KM) curve was applied to analyze the overall survival (OS) and disease-specific survival (DSS). The immune landscapes of CD93 were analyzed by the SangerBox (http://sangerbox.com/) and immunedeconv package. The immune score is a sophisticated tissue-based assay that defines the score by precisely quantifying and identifying T lymphocytes infiltrating the tumor in specific regions. The stromal score, which captures the presence of stroma in the TME, uses expression data for specific gene signatures associated with the stromal component of the TME to predict levels of infiltrating stromal cells. Estimated scores are used to infer tumor purity in the TME. These scores were calculated by the SangerBox using the ESTIMATE algorithm. The correlation between CD93 levels and other gene mutation status in these cancers was analyzed by the MuTarget dataset (https://www.mutarget.com/analysis?type=target) (31). The Gene set variation analysis algorithm (GSVA) (32), Kyoto Encyclopedia of Genes and Genomes database (KEGG; https://www.genome.jp/kegg/) (33, 34), and HALLMARK database were applied to identified enriched signaling pathways. The survival difference between mutant and WT, CNV groups, and methylation were analyzed by the GSCA dataset. The TIDE (http://tide.dfci.harvard.edu) and TISMO (http://tismo.cistrome.org) websites were used to analyze the immunotherapy and gene treatment responses of CD93 in these cancers.. The Gene Set Cancer Analysis (GSCA; http://bioinfo.life.hust.edu.cn/GSCA/#/), CCLE, and CellMiner (https://discover.nci.nih.gov/cellminer/) (35) datasets were used to predict the sensitive small molecule drugs. The correlation of CD93 expression with treatment response in breast cancer, OV, GBM, and colorectal cancer was predicted by the ROC Plotter (http://www.rocplot.org/site/index) (36, 37).

### Single-cell sequencing analysis

The R package (Seurat) were applied for BRCA and STAD data integration and quality control (38). Dimensionality reduction using Principal Component Analysis (PCA). Visualization of CD93 expression by R packages (Vlnplot, Dimplot, and Featureplot). The FindClusters function was applied to cluster the cells together. Identification of tumor cells by the R package (infercnv and copycat). Visualization of dimensionality reduction with UMAP functions.

### Multiple immunofluorescence staining

Multiple immunofluorescence staining was performed as previously described (39, 40). The primary Abs were CD8 (Mouse, 1:3000, Proteintech), CD68 (Rabbit, 1:3000, AiFang biological), CD93 (Rabbit, 1:200, Thermo Fisher), and CD163 (Rabbit, 1:3000, Proteintech). PV6001 (horseradish peroxidase-conjugated secondary antibody, ZSGB-BIO, China) was the secondary antibody, and the tyramide signal was amplified to TSA [FITC-TSA, CY3-TSA, 594-TSA, and 647-TSA (Servicebio, China)]. Image analysis and positive cell quantification were performed by Caseviewer (C.V 2.3, C.V 2.0) and Pannoramic viewer (P.V 1.15.3). Negative controls excluded the primary Ab. We obtained the tissue microarray (HOrg-C110PT-01) from the Outdo Biotech company (Shanghai, China), and the ethics were approved.

### Statistical analysis

The R package calculated the optimal cutoff of CD93 (survminer). A student's t-test (normally distributed data) and Kruskal-Wallis's test (non-normally distributed data ) compared CD93 expression in the cancer and corresponding samples, respectively. Meanwhile, the log-rank test was applied to explore the prognostic role of CD93. All tests were bilateral, and P< 0.05 was set as statistically significant.

### Expression and prognostic value of CD93

**Figure 1:**
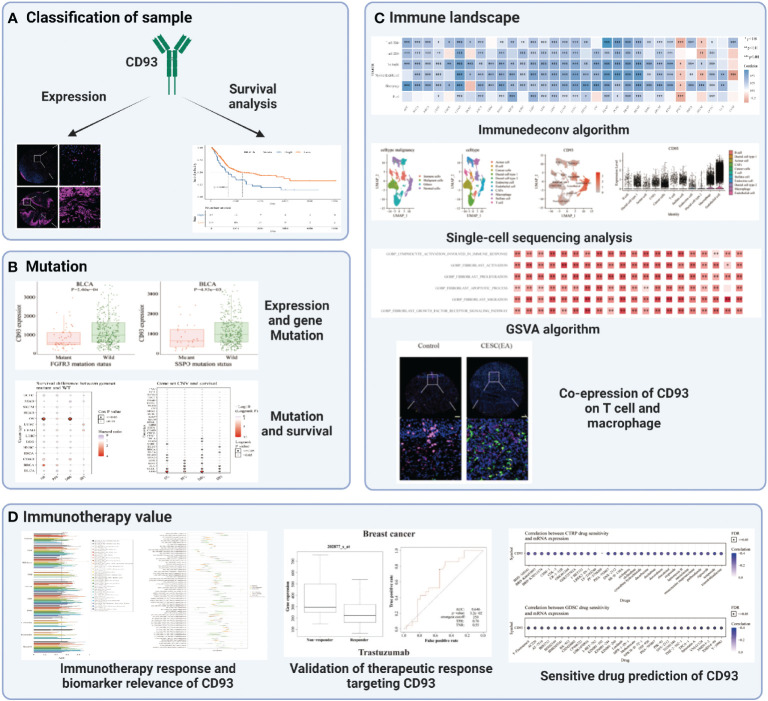
The flow chart of this study. *p < 0.05, **p < 0.01, ***p < 0.001.

**Figure 2:**
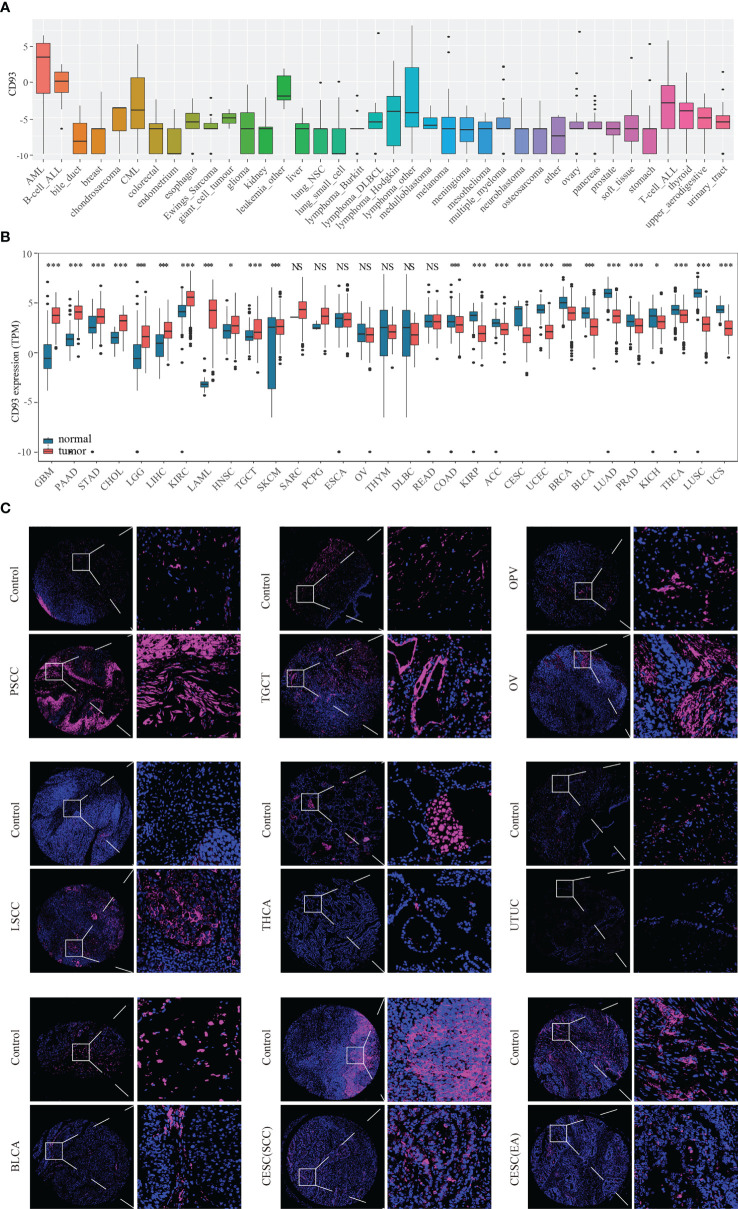
CD93 levels in tumor samples and counterparts. CD93 mRNA expression in cancer cell lines from the CCLE dataset **(A)**. CD93 mRNA expression in cancer and normal samples from the TCGA and GETX datasets **(B)**. CD93 protein expression in cancer and control samples in tumor microarrays **(C)**. *p < 0.05, ***p < 0.001, NS, no significant differences.

The flow chart designed in this paper is shown in **Figure 1**. Firstly, we used the CCLE, TCGA, and GTEX databases to explore the CD93 levels in the cancers and their counterparts. Data from the CCLE database showing CD93 expression in tumor cell lines, of which the top three cell lines were AML, B cell ALL, and leukemia (**Figure 2A**). Elevated mRNA levels of CD93 in tumor samples compared to normal controls in GBM, PAAD, STAD, CHOL, LGG, LIHC, KIRC, acute myeloid leukemia (LAML), HNSC, TGCT, and SKCM (**Figure 2B**; P<0.05). Conversely, decreased mRNA levels of CD93 in tumor samples compared to normal controls in COAD, KIRP, ACC, CESC, UCEC, BRCA, BLCA, LUAD, PRAD, KICH, THCA, LUSC, and UCS (**Figure 2B**; P<0.05). Meanwhile, we verified the protein levels of CD93 in our tumor microarrays through immunofluorescence staining (**Figure 2C**). Data showed that CD93 protein levels were upregulated in the tumor samples compared to control (pericancerous) samples in penile squamous cell carcinoma (PSCC), laryngeal squamous cell carcinoma (LSCC), and TGCT. Meanwhile, CD93 protein levels were decreased in the tumor samples than in control samples in THCA, UTUC, BLCA, and CESC. The CD93 protein levels were also expressed in ovarian serous papillary adenocarcinoma (OPV) and OV.

In addition, the prognostic role of CD93 in these cancers was explored using the KM algorithm. Results show that CD93 has a good value in predicting OS (**Supplementary Figure 1A**) and DSS (**Supplementary Figure 1B**) in multiple cancers. Low CD93 mRNA levels were associated with longer OS in BLCA, CESC, COAD, ESCA, GBM, UCEC, UVM, KIRP, LAML, LGG, LIHC, READ, LUSC, MESO, OV, STAD, and THCA (**Supplementary Figure 1C**; P<0.05). Conversely, low CD93 mRNA levels were associated with shorter OS in PCPG, LUAD, SARC, HNSC, and KIRC (**Supplementary Figure 1C**; P<0.05). In addition, low CD93 mRNA levels related to better DSS in BLCA, COAD, ESCA, LGG, LUSC, KIRP, MESO, OV, UCEC, and UVM, and related to poor DSS in HNSC, LUAD, KIRC, SARC, and PCPG (**Supplementary Figure 2A**; P<0.05). A summary of CD93 expression in pan-cancer and its association with prognosis is shown in **Supplementary Figure 2B**.

### Mutation analysis of CD93 in pan-cancer

**Figure 3:**
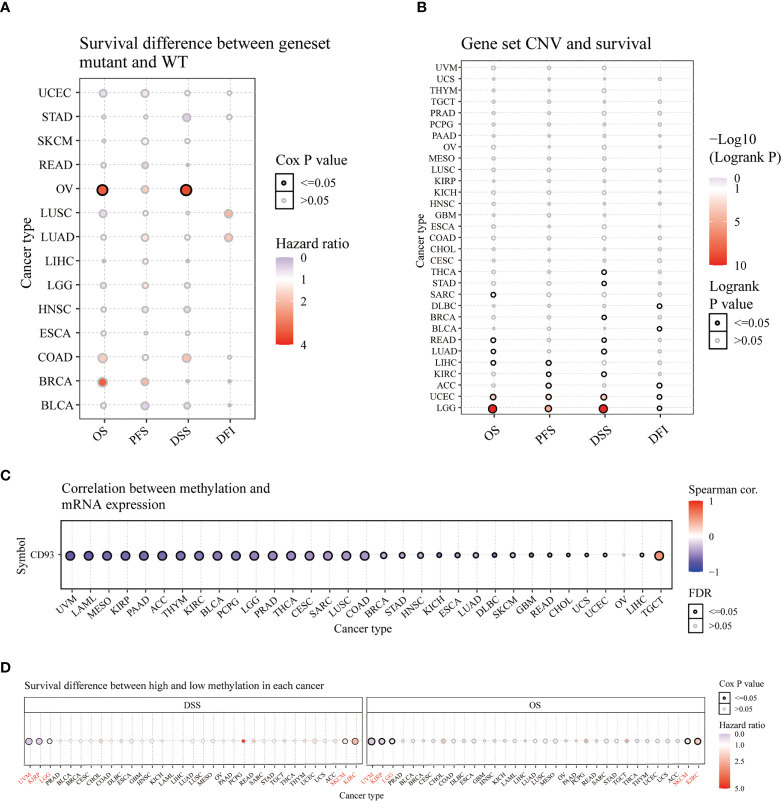
Survival analysis of CD93 in pan-cancer from the GSCA database. Survival difference between CD93 mutant and WT **(A)**. CD93 CNV and survival **(B)**. Correlation between CD93 methylation and mRNA expression **(C)**. Survival difference between CD93 high and low methylation **(D)**.

Furthermore, we analyzed the survival difference between CD93 genome mutants and the WT group in these cancers through progression-free survival (PFS), disease-free interval (DFI), OS, and DSS analysis (**Figure 3A**). The DSS and OS in the CD93 mutant group significantly differ from the WT group in OV (**Figure 3A** and **Table S1**; P<0.05). CD93 copy number variation (CNV) and survival analysis results showed significant differences in OS for LGG, UCEC, READ, LIHC, SARC, and LUAD (P<0.05); in PFS for LGG, UCEC, ACC, LIHC, and KIRC (P<0.05); in DSS for LGG, UCEC, LUAD, KIRC, THCA, STAD, READ and BRCA (P<0.05); in DFI for ACC, UCEC, DLBC, LGG and BLCA (**Figure 3B** and **Table S2**; P<0.05). CD93 RNA expression is associated with methylation in almost every type of cancer except OV (**Figure 3C** and **Table S3**; P<0.05). CD93 high methylation group showed a significant difference with the low methylation group in DSS and OS for UVM, KIRP, LGG, SKCM, and KIRC (**Figure 3D** and **Table S4**; P<0.05).

**Figure 4:**
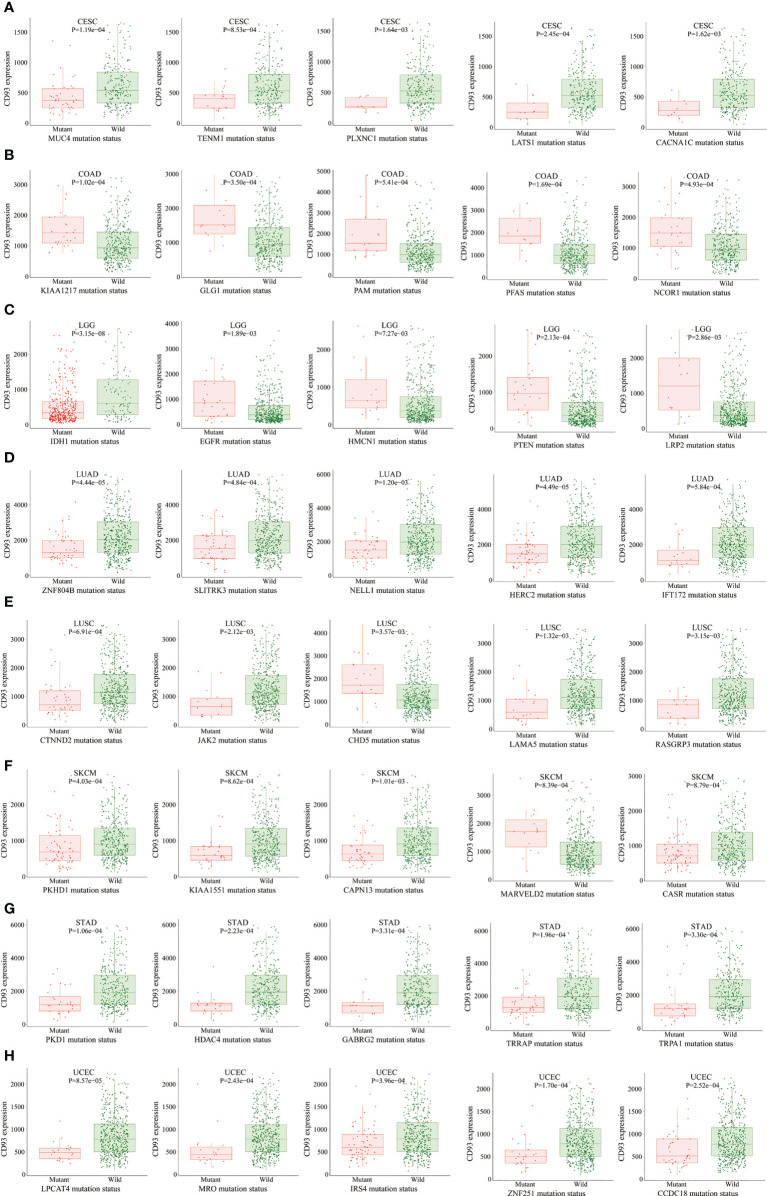
The correlation between CD93 expression and gene mutation status. CESC **(A)**, COAD **(B)**, LGG **(C)**, LUAD **(D)**, LUSC **(E)**, SKCM **(F)**, STAD **(G)**, and UCEC **(H)**.

The MuTarget dataset showed that CD93 expression was associated with several gene mutational states in CESC, including MUC4, TENM1, PLXNC1, LATS1, and CACNA1C. The wild group of these genes has more CD93 expression than the mutant group (**Figure 4A**; P<0.01). In COAD, the mutant group of KIAA1217, GLG1, PAM, PFAS, and NCOR1 has more CD93 expression than the wild group (**Figure 4B**; P<0.001). In LGG, the mutant group of EGFR, HMCN1, PTEN, and LRP2 has more CD93 expression than the wild group, while the wild group of IDH1 has more CD93 expression than the mutant group (**Figure 4C**; P<0.01). In LUAD, the wild group of ZNF804B, SLITRK3, NELL1, HERC2, and IFT172 has more CD93 expression than the mutant group (**Figure 4D**; P<0.01). In LUSC, the wild group of CTNND2, JAK2, LAMA5, and RASGRP3 has more CD93 expression than the mutant group, while the mutant group of CHD5 has more CD93 expression than the wild group (**Figure 4E**; P<0.01). In SKCM, the wild group of PKHD1, KIAA1551, CAPN13, and CASR has more CD93 expression than the mutant group, while the mutant group of MARVELD2 has more CD93 expression than the wild group (**Figure 4F**; P<0.01). In STAD, the wild group of PKD1, HDAC4, GABRG2, TRRAP, and TRPA1 has more CD93 expression than the mutant group (**Figure 4G**; P<0.01). In UCEC, the wild group of LPCAT4, MRO, IRS4, ZNF251, and CCDC18 has more CD93 expression than the mutant group (**Figure 4H**; P<0.001). In BLCA, the wild group of FGFR3, SSPO, and KHDRBS2 has more CD93 expression than the mutant group, while the mutant group of KDM5B has more CD93 expression than the wile group (**Supplementary Figure 3A**; P<0.01). In SARC, the wild group of AHNAK and CCDC168 has more CD93 expression than the mutant group (**Supplementary Figure 3B**; P<0.01). In LIHC, the wild group of CTNNB1, KMT2D, and AXIN1 has more CD93 expression than the mutant group, while the mutant group of COL6A3 has more CD93 expression than the wile group (**Supplementary Figure 3C**; P<0.01). In OV, the mutant group of AOC2, DNAH11, ZNF835, and PLEKHG1 has more CD93 expression than the wild group (**Supplementary Figure 3D**; P<0.01). In HNSC, the wild group of AJUBA has more CD93 expression than the mutant group (**Supplementary Figure 3E**; P<0.01). In multiple myeloma, the wild group of SLC22A3, PTOV1, GRM2, and NBPF10 has more CD93 expression than the mutant group, while the mutant group of CXXC1 has more CD93 expression than the wile group (**Supplementary Figure 3F**; P<0.001).

### Immune characteristics of CD93 in the tumor microenvironment

Next, to clarify the immune characteristics of CD93 in the TME in these cancers, we explored the relationship between CD93 expression and three scores (immune, estimate, and stromal) in 33 cancers using the ESTIMATE algorithm through the SangerBox website. CD93 expression positively correlated with immune scores in almost all cancers, except DLBC, KIRC, TGCT, THCA, and THYM (**Supplementary Figure 4**; P<0.05). CD93 levels were strongly correlated with estimate scores for almost all cancers except TGCT and THCA (**Supplementary Figure 5**; P<0.01). High CD93 expression was strongly associated with high stromal scores in all cancers (**Supplementary Figure 6**; P<0.01). In addition, six immune infiltration algorithms were applied, including EPIC, TIMER, QUANTIAEQ, xCell, MCPCOUNTER, and CIBERSORT (**Supplementary Figure 7**) to analyze the correlation between CD93 and stromal cells in these cancers. The results indicated that CD93 was closely associated with these stromal cells in the TME of these cancers. Results indicated that CD93 is closely related to these stromal cells in the TME in these cancers. Especially, high CD93 levels were positively correlated with the expression of endothelial cells, T cells (CD8^+^ and CD4^+^), neutrophils, myeloid dendritic cells, macrophages, B cells, T cell regulatory (Tregs), M1 and M2 macrophages, monocytes, and hematopoietic stem cells in almost all cancers. On the contrary, the expression of CD4^+^ T cell (Th1 and central memory), T cell follicular helper, B cell plasma, and activated NK cell was negatively correlated with CD93 levels.

Neoantigens, a group of tumor-specific antigens generated by tumor cell mutations in the TME, have the potential to become valuable targets for tumor immunotherapy (41, 42). The expression of neoantigens kept a close correlation with CD93 levels in BRCA, STAD, CESC, THCA, and LGG (**Supplementary Figure 8**; P<0.05). Moreover, we studied the correlation between CD93 and other classical immune checkpoints in these cancers. Results showed that CD93 expression was closely associated with the levels of several immune checkpoints in many cancers, including NRP1, LAIR1, CD28, CD200R1, HAVCR2, CD276, VSIR, and CD86 (**Supplementary Figure 9**; P<0.05).

### Functional analysis based on CD93 expression

**Figure 5:**
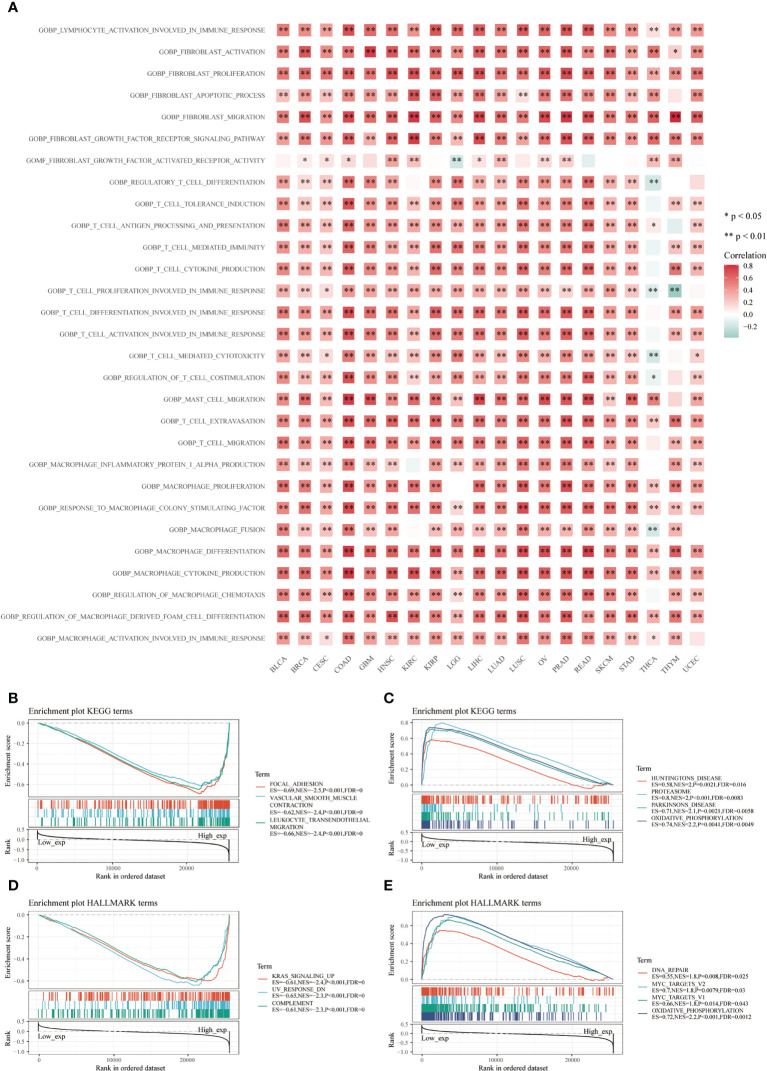
Functional analysis based on CD93 expression. Correlation analysis of CD93 from the GSVA algorithm **(A)**. Top three negative **(B)** and top four positive **(C)** enriched pathways using the KEGG database. The top three negative **(D)** and four positive **(E)** enriched pathways using the HALLMARK database.

Many immune-related pathways based on the GSVA algorithm were significantly positively correlated with CD93 expressions in these cancers, such as lymphocyte activation involved in immune response, fibroblast activation and proliferation, fibroblast migration, fibroblast growth factor receptor signaling pathway, T cell extravasation, response to macrophage colony-stimulating factor, macrophage cytokine production, macrophage and macrophage-derived foam cell differentiation (**Figure 5A**; P<0.05). These signaling pathways play an irreplaceable role in tumor immunity in the TME, especially in the infiltration and activation of T cells, CAFs, macrophages, and mast cells. Additionally, the top three negatively enriched pathways were focal adhesion, vascular smooth muscle contraction, and leukocyte transendothelial migration (**Figure 5B**; P<0.001), while the top four positively enriched pathways were Huntington’s disease, proteasome, Parkinson’s disease, and oxidative phosphorylation analyzed from the KEGG database (**Figure 5C**; P<0.01). KRAS signaling up, UV response DN, and complement were the top three negatively enriched pathways (**Figure 5D**; P<0.001), while DNA repair, MYC targets V2, MYC targets V1, and oxidative phosphorylation were the top four positively enriched pathways analyzed from the HALLMARK database (**Figure 5E**; P<0.05).

### Relationship between CD93 and tumor and stromal cells analyzed by the single-cell sequencing and multiplex immunofluorescence staining

**Figure 6:**
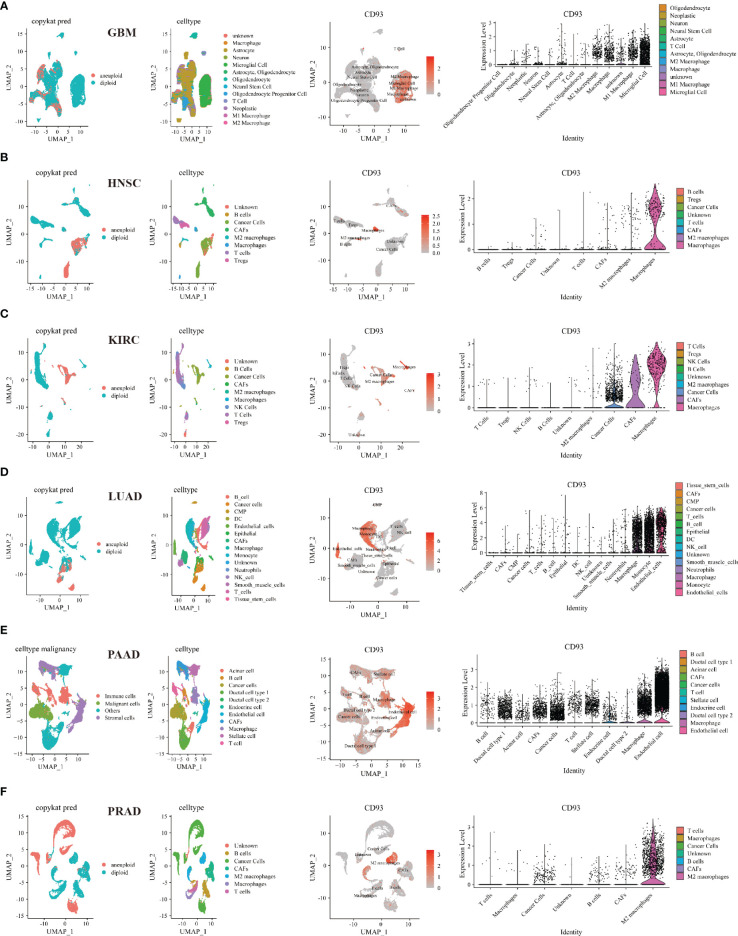
Single-cell sequencing analyzing the co-expression of CD93 on tumor and stromal cells in the TME. Co-expression of CD93 on tumor and stromal cells in GBM **(A)**, HNSC **(B)**, KIRC **(C)**, LUAD **(D)**, PAAD **(E)**, and PRAD **(F)**.

Then, we investigated the relationship between CD93 and tumor and stromal cells in the TME of these cancers, including GBM, HNSC, KIRC, LUAD, PAAD, PRAD, BLCA, BRCA, CHOL, COAD, LIHC, OV, SKCM, and STAD (**Figure 6** and **Supplementary Figure 10**). Interestingly, microglial cells, M1 and M2 macrophages, macrophages, B cells, NK cells, astrocyte, neurons, neoplastic, oligodendrocyte, CAFs, T cells, endothelial cells, monocyte, neutrophils, smooth muscle cells, epithelial, cancer cells, stellate cells, endocrine cells, acinar cells, ductal cell type 1, TEC, and HPC-like were found to express CD93 in these cancers.

**Figure 7:**
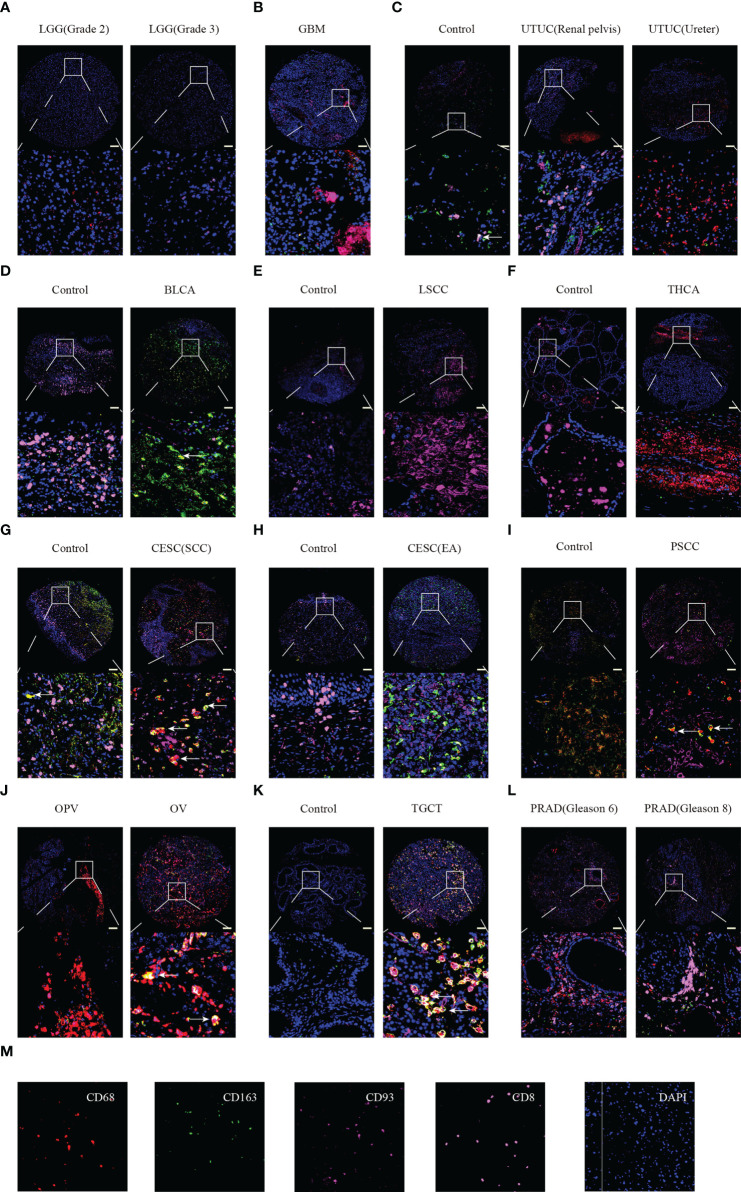
Multiplex immunofluorescence staining identified the relationship between CD93 expression and CD8, CD68, and CD163 in tumor and normal samples. LGG **(A)**, GBM **(B)**, UTUC **(C)**, BLCA **(D)**, LSCC **(E)**, THCA **(F)**, CESC **(G, H)**, PSCC **(I)**, OPV and OV **(J)**, TGCT **(K)**, and PRAD **(L)**. Staining for CD8, CD163, CD93, CD9, and DAPI **(M)**. Scale bar=100um.

Furthermore, we performed multiplex immunofluorescence staining to identify the relationship between CD93 and CD8 (marker for T cells), CD68 (marker for macrophages), and CD163 (marker for M2 macrophages) in these cancers. CD8 is labeled in pink, CD68 is labeled in red, CD163 is labeled in green, CD93 is labeled in rose red, and DAPI is labeled in blue (**Figure 7M**). WHOIII gliomas have higher CD93 expression than WHOII gliomas (**Figure 7A**). CD93 was found to be closely related to CD8 in GBM (**Figure 7B**), UTUC (**Figure 7C**), and PRAD (**Figure 7L**). CD93 was found to be closely related to CD68 in UTUC (**Figure 7C**), THCA (**Figure 7F**), CESC (SCC) (**Figure 7G**), PSCC (**Figure 7I**), OPV, and OV (**Figure 7J**), TGCT (**Figure 7K**), and PRAD (**Figure 7L**). In addition, CD163 was found to be closely related to CD93 expression in BLCA (**Figure 7D**), CESC (**Figures 7G, H**), and TGCT (**Figure 7K**). LSCC expressed more CD93 levels than the control (**Figure 7E**). Interestingly, CD93 protein levels appear to be higher in GBM than in LGG.

### Prediction of tumor immunotherapy value based on CD93 expression

**Figure 8:**
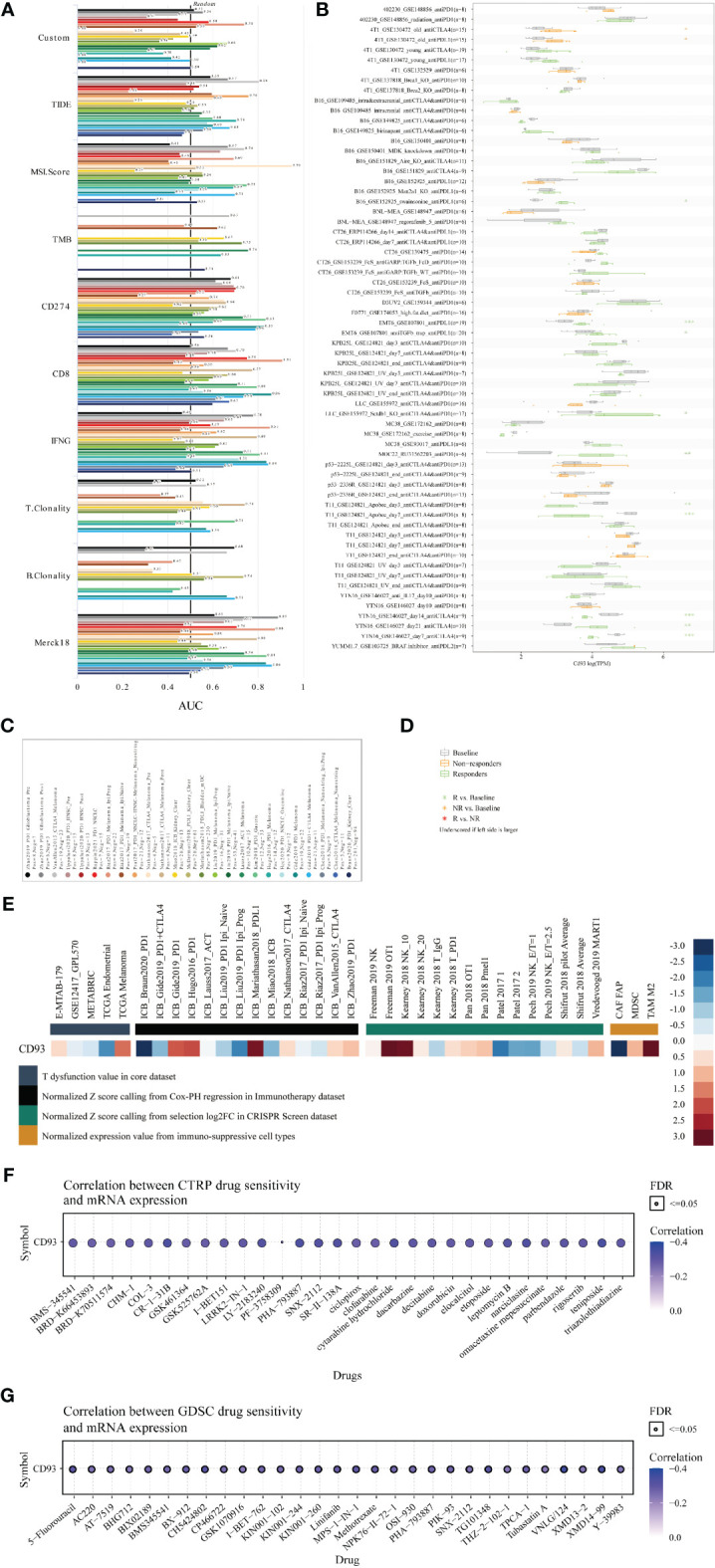
Immunotherapy value and sensitive drug prediction of CD93 based on the public datasets. Immunotherapy response **(A)** and biomarker relevance **(B)** of CD93 in immunotherapy cohorts. Notes for the immunotherapy response **(C)** and biomarker relevance **(D)** of CD93.Therapeutic responses of CD93 in mechanistic follow-up experiments in the given datasets **(E)**. Sensitive small compounds predicted from the CTRP **(F)** and GDSC **(G)** websites based on CD93 levels.

Finally, to systematically clarify the underlying value of CD93 as an immunotherapy target in these cancers, we predicted the immunotherapy response and sensitive drugs from the public databases (**Figure 8**). Based on the predictive role of classical biomarkers for response outcomes and OS in human immunotherapy cohorts, we calculated the biomarker correlation of CD93 by comparing it to these classical biomarkers. Of the total 25 immunotherapy cohorts, CD93 alone showed an AUC above 0.5 in 8 immunotherapy cohorts (**Figures 8A, C**). The predictive value of CD93 was higher than the B. Clonality with AUC value above 0.5 in seven immunotherapy cohorts. CD93 had the same predictive value as the TMB with an AUC value above 0.5 in 8 immunotherapy cohorts. The predictive value of CD93 was lower than the T.Clonality, MSI score, IFNG, and CD8, TIDE, and CD274, with AUC values above 0.5 in 9, 13, 17, 18, 18, and 21 immunotherapy cohorts, respectively. CD93 significantly predicted immunotherapy response in 4 immunotherapy cohorts, where responders were more likely to have high CD93 levels (**Figures 8B, D**). The ROC Plotter dataset analyzed the prediction of response to therapy targeting CD93 in breast, OV, GBM, and colorectal cancer. Results showed that the AUC of treatment with trastuzumab in breast cancer was 0.646 (**Supplementary Figure 11A**; P<0.05). The AUC of treatment with capecitabine in colorectal carcinoma was 0.677 (**Supplementary Figure 11B**; P<0.05). Therapeutic responses of CD93 in mechanistic follow-up experiments in the core dataset, immunotherapy dataset, CRISPR Screen dataset, and immune-suppressive cell types were predicted from the TIDE website (**Figure 8E**).

Then, we predicted the sensitive small molecule drugs through CTRP (**Figure 8F**) and GDSC (**Figure 8G**) datasets. The top five sensitive drugs were BRD-K30748066, isoliquiritigenin, teniposide, PHA-793887, and CR-1-31B based on CD93 expression from the CTRP dataset (**Table S5**; P<0.05). The top five sensitive drugs were VNLG/124, XMD14-99, CH5424802, TG101348, and 5-Fluorouracil based on CD93 expression from the GDSC dataset (**Table S5**; P<0.0001). The top three sensitive compounds were PF2341066, PD-0332991, and L-685458 based on CD93 expression from the CCLE dataset (**Table S6**; P<0.05). Triostin a, sb-253226, toxin. delta.53l, pederin, and euserotin were top-five sensitive compounds based on CD93 expression from the CellMiner dataset. In contrast, 2-[(3,4-dicloro) anilino]-3-phenyl-5,7-diamino quinoxaline, janus red, juncusol deriv (compound 1), 1,4-benzenediamine (9ci), n-butyl-n’-phenyl-, and 1-benzyl-3-hexadecyl-2-methylimidazolium chloride were top-five sensitive compounds based on CD93 methylation from the CellMiner dataset (**Table S6**; P<0.05). These small molecules have been found to play anti-tumor roles in several cancers. For example, CR-1-31B, a synthetic rocaglate and a potent eIF4A inhibitor, significantly reduced the growth and apoptosis of neuroblastoma and gallbladder tumor cells (43, 44). PHA-793887, a novel and potent inhibitor of CDK, showed promising efficacy in the human ovarian A2780, colon HCT-116, and pancreatic BX-PC3 cancer xenograft models (45, 46). VNLG/124 exhibited potent anti-proliferative effects in both hormone-insensitive/drug-resistant breast cancer cell lines and the hormone-insensitive PC-3 prostate cancer cell lines (47). Computational models for drug sensitivity prediction indicated XMD14-99 could act as a kinase inhibitor to exert efficacy in several cancer cell lines (47). Excitingly, our results indicated that CD93 expression is associated with prognosis. These results provide a theoretical basis for our preclinical and clinical cancer experiments targeting CD93 expression in the TME. However, there are also some limitations in our study. First, due to the relatively small samples of certain cancer types on the tissue microarray, it is difficult for us to quantify and perform statistical analyses. Second, the exact relationship between CD93 and tumor immunity and related signaling pathways in these cancers were not revealed through *in vivo* and *in vitro* study, more validated experiments are needed in the future.

## Discussion

The previous study has demonstrated the critical role of CD93 in tumor vascularization and upregulated CD93 in tumor vessels as a potential malignant biomarker in several cancers. Given that the molecular characteristics of CD93 in pan-cancer remain unexplored, we performed large-scale single-cell and bulk sequencing analysis to identify the prognostic value and immune features of CD93 in these cancers. In our paper, we observed the mRNA expression of CD93 in 38 cancer cell lines, 31 cancer samples, and counterparts based on public databases. By combining TCGA and GTEX datasets, CD93 levels were higher in GBM, PAAD, STAD, CHOL, LGG, LIHC, KIRC, LAML, HNSC, TGCT, and SKCM than in normal controls. CD93 levels were lower in COAD, KIRP, ACC, CESC, UCEC, BRCA, BLCA, LUAD, PRAD, KICH, THCA, LUSC, and UCS than in controls. At the same time, the immunofluorescence staining showed that CD93 protein was upregulated in PSCC, LSCC, and TGCT than control samples while downregulated in THCA, UTUC, BLCA, and CESC than control samples. CD93 protein levels appear to be higher in GBM than in LGG. In addition, CD93 can serve as a stable prognostic biomarker in almost all cancers except ACC, BRCA, CHOL, DLBC, PAAD, PRAD, SKCM, and UCS. The survival difference between CD93 mutants and WT, CNV of CD93 and survival, the correlation between CD93 methylation and mRNA levels, and the survival difference between CD93 low and high methylation in pan-cancer were analyzed.

The pathological process of tumor formation, growth, and migration is regulated by genetic mutations in tumor cells and the dynamic interactions of the components in the TME (48, 49). Cancer cells, stromal cells, vascular system and extracellular matrix such as fibronectin, laminin, enzymes, and glycoproteins together form a complex and interconnected tumor microenvironment (50, 51). The tumor stromal cells are composed of CAFs, adipocytes, endothelial cells, pericytes, and immune cells, including M1 and M2 macrophages, T cells, B cells, neutrophils, microglia, monocyte, and NK cells (52, 53). These stromal cells play a crucial role in developing tumor, metastasis, immune infiltration, and chemoresistance by producing growth factors, cytokines, chemokines, pro-tumorigenic and anti-tumorigenic factors (54, 55). For example, CAFs are essential for the TME to remodel the extracellular matrix and mediate leukocyte infiltration (56). Increasing evidence showed that activated CAFs could regulate tumor cell invasion and growth by secreting soluble factors (exosomes, HGF and GAS6) and depleting metabolic factors (lactate, amino acid, alanine, and aspartate) (57). Meanwhile, CAFs induced the infiltration and activation of macrophages, endothelial cells, and T cells by producing VEGF, TGFβ, IL-6, CCL2, and CXCL12. Cancer-associated endothelial cells play an irreplaceable role in tumor cell growth and migration. A study in breast cancer treatment found that cancer-associated endothelial cells contribute to the production of CXCL1/2 and S100A8/9, which eventually led to breast cancer cell survival and drug resistance (58). Macrophages have the function of phagocytosing and digesting foreign antigens, and play a crucial role in clearing away cellular debris and tumor cells (59). Tumor-associated macrophages (M2) lose their ability to kill tumors due to the absence of phagocytosis, which eventually leads to the spread of tumor cells to other tissues and organs (60). The intratumoral T cells, a significant component of the infiltrated immune cells in the TME, comprises CD4^+^, CD8^+^, naïve, memory, effector, and regulatory T cells. These different T cell subtypes are essential mediators of anti-tumor immunity, recognizing and responding to tumor-expressed antigens (61).

In current paper, we observed the co-expression of CD93 on tumor and stromal cells based on bulk and large-scale single-cell sequencing and tumor chips. Many stromal cells expressed high CD93 levels in the tumor microenvironment of pan-cancer, such as endothelial cells, B cells, T cells, neutrophils, myeloid dendritic cells, macrophages, monocyte, and hematopoietic stem cell. Notably, CD93 expression significantly correlated with these scores (immune, stromal, and estimate) in almost all cancers. Moreover, large-scale single-cell sequencing analysis demonstrated that macrophages, astrocytes, CAFs, T cells, B cells, endothelial cells, neutrophils, and cancer cells are the primary cells that expressed CD93 in the TME. Furthermore, the relationship between CD8, CD68, and CD163 in these cancers was verified by multiplex immunofluorescence staining. Furthermore, the functional signaling analysis indicated that many tumor immune-related pathways were enriched according to CD93 expression, such as immune cells (fibroblast, macrophages, and T cells) activation and migration, focal adhesion, leukocyte transendothelial migration, oxidative phosphorylation, and complement. These results invariably illustrate the pivotal role of CD93 in tumor immunity.

Immunotherapy, focusing on inhibiting immune checkpoints, has undoubtedly been the highest achievement of cancer treatment in the last decade. The programmed death 1 (PD-1)/programmed cell death-ligand 1 (PD-L1) and cytotoxic T-lymphocyte–associated antigen 4 (CTLA-4)/B7 are two classical immune checkpoint signaling pathways, which negatively mediate T cell immunity during the activation and proliferation of T cells under pathological conditions. Targeting PD1/PD-L1 and CTLA-4/B7 with specific inhibitors has demonstrated exciting preclinical and clinical efficacy in several cancers. This paper studied the correlation between CD93 and other classical immune checkpoints in pan-cancer. Data showed that the many immune checkpoints positively correlate with CD93 expression in many cancers, particularly NRP1, LAIR1, VSIR, and CD86. Predicting the immunotherapy value and the optimal individualized therapeutic drugs from public databases and computational models has been increasingly attractive in recent years (62–64). Biomarker correlations for CD93 were calculated in more than 20 immunotherapy cohorts to validate its predictive value. Out of a total of 25 immunotherapy cohorts, eight immunotherapy cohorts had AUC values above 0.5. The predictive value of CD93 was higher than the B.Clonality with AUC value above 0.5 in 7 immunotherapy cohorts. CD93 had the same predictive value as the TMB with an AUC value above 0.5 in 8 immunotherapy cohorts. Moreover, the predictive value of CD93 was lower than the T.Clonality, MSI score, IFNG and CD8, TIDE and CD274, with AUC values above 0.5 in 9, 13, 17, 18, 18 and 21 immunotherapy cohorts, respective. GDSC and CTRP are free, publicly available databases of over 500 small molecule compounds based on the therapeutic response of over 1000 genetically characterised human cancer cell lines. Ultimately, we discovered a lot of sensitive small molecules according to CD93 levels from the public datasets, such as CR-1-31B, PHA-793887, SR-II-138A, cytarabine hydrochloride, narciclasine, VNLG/124, XMD14-99, TG101348, CH5424802, and 5-Fluorouracil. These data will provide theoretical support for future clinical trials targeting CD93 in these cancers.

## Conclusion

In this project, we comprehensively analyzed the prognostic value and immune profile of CD93 in pan-cancer using large-scale single-cell and bulk sequencing analysis. CD93 is highly involved in tumor immunity and may act as a novel immune checkpoint in immunotherapy of these cancers. Therefore, therapeutic strategies that block CD93 in the tumor microenvironment are expected to benefit patients with malignancies.

## Data availability statement

The original contributions presented in the study are included in the article/**Supplementary Material**. Further inquiries can be directed to the corresponding authors.

## Author contributions

Original Draft, Methodology, Validation, Visualization: JWZ and HZ. Data Curation, Validation: AG, WW, YP, PL, and JZ. Investigation: ZL and YT. Conceptualization, Methodology, Supervision, Project Administration, and Funding Acquisition: HZ, and QC. All authors contributed to the article and approved the submitted version.

## Funding

This work was supported by the Hunan Provincial Health Committee Foundation of China (NO.202204044869), Hunan Provincial Natural Science Foundation of China (NO.2020JJ5850).

## Acknowledgments

The author express gratitude to the public databases, websites, and softwares used in the paper. We are grateful to the High Performance Computing Center of Central South University for partial support of this work.

## Conflict of interest

The authors declare that the research was conducted in the absence of any commercial or financial relationships that could be construed as a potential conflict of interest.

## Publisher’s note

All claims expressed in this article are solely those of the authors and do not necessarily represent those of their affiliated organizations, or those of the publisher, the editors and the reviewers. Any product that may be evaluated in this article, or claim that may be made by its manufacturer, is not guaranteed or endorsed by the publisher.
