# Supplementary material for: Identify the immune characteristics and immunotherapy value of CD93 in the pan-cancer based on the public data sets
Source: Front Immunol. 2022 Oct 27;13:907182. doi: 10.3389/fimmu.2022.907182 (PMC9646793; doi:10.3389/fimmu.2022.907182)
Supplement: Supplementary file 1 [file DataSheet_1.docx]

**Supplementary Figures**

**
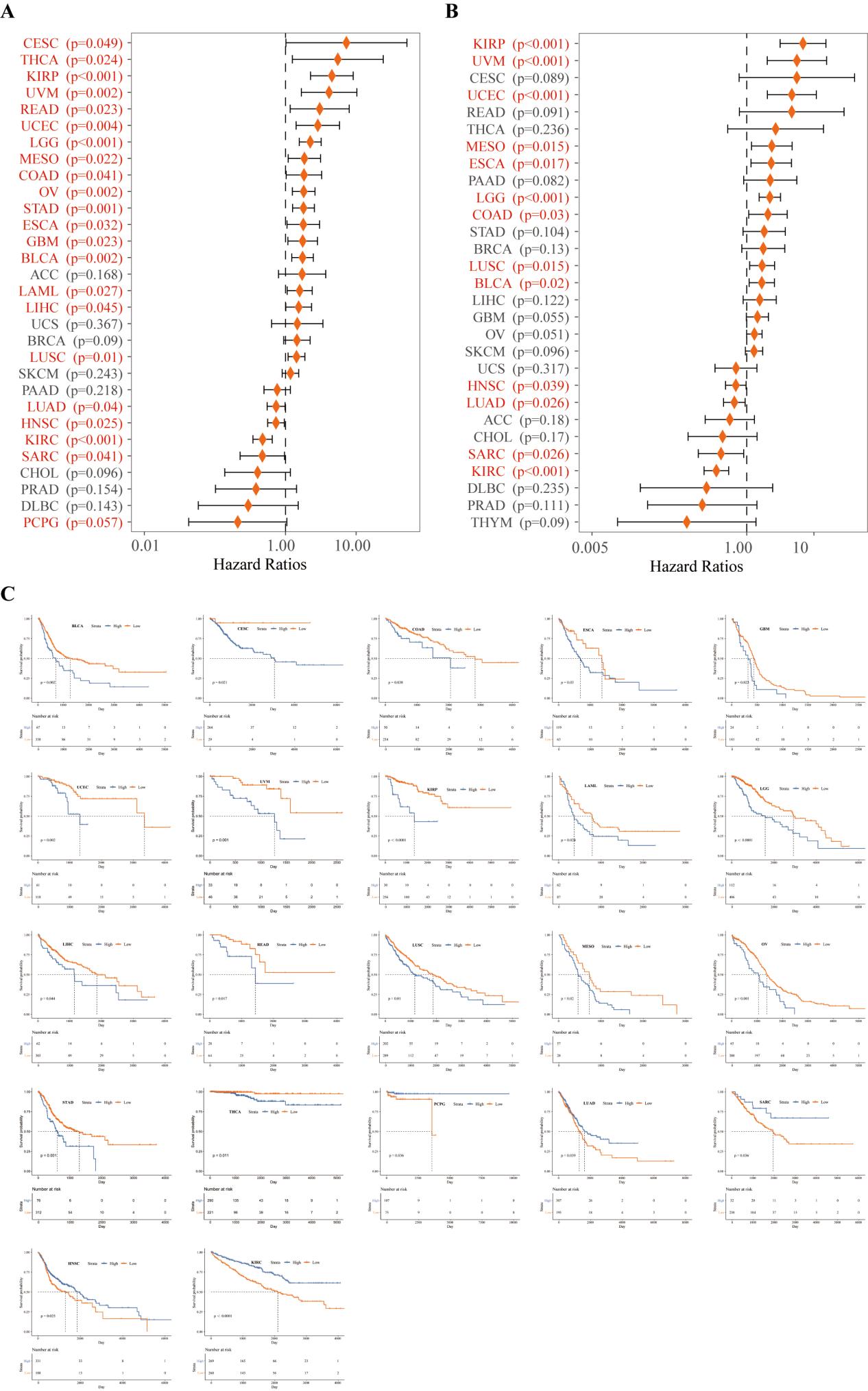
Figure S1. Prognostic value CD93 in pan-cancer from the TCGA database. Survival analysis of CD93 on OS (A) and DSS (B) displayed by the forest plot. Adrenocortical carcinoma (ACC), bladder cancer (BLCA), breast carcinoma (BRCA), cervical cancer (CESC), cholangiocarcinoma (CHOL), colorectal cancer (COAD), esophageal cancer (ESCA), glioblastoma (GBM), head and neck squamous cell carcinoma (HNSC), kidney chromophobe (KICH), kidney renal clear cell carcinoma (KIRC), kidney renal papillary cell carcinoma (KIRP), low-grade glioma (LGG), liver hepatocellular carcinoma (LIHC), lung adenocarcinoma (LUAD), lung squamous cell carcinoma (LUSC), mesothelioma (MESO), ovarian serous cystadenocarcinoma (OV), pancreatic adenocarcinoma (PAAD), pheochromocytoma and paraganglioma (PCPG), prostate cancer (PRAD), rectal cancer (READ), sarcoma (SARC), skin cutaneous melanoma (SKCM), stomach adenocarcinoma (STAD), testicular cancer (TGCT), thyroid carcinoma (THCA), thymoma (THYM), uterine corpus endometrial carcinoma (UCEC), uterine carcinosarcoma (UCS) and ocular melanomas (UVM).**

**
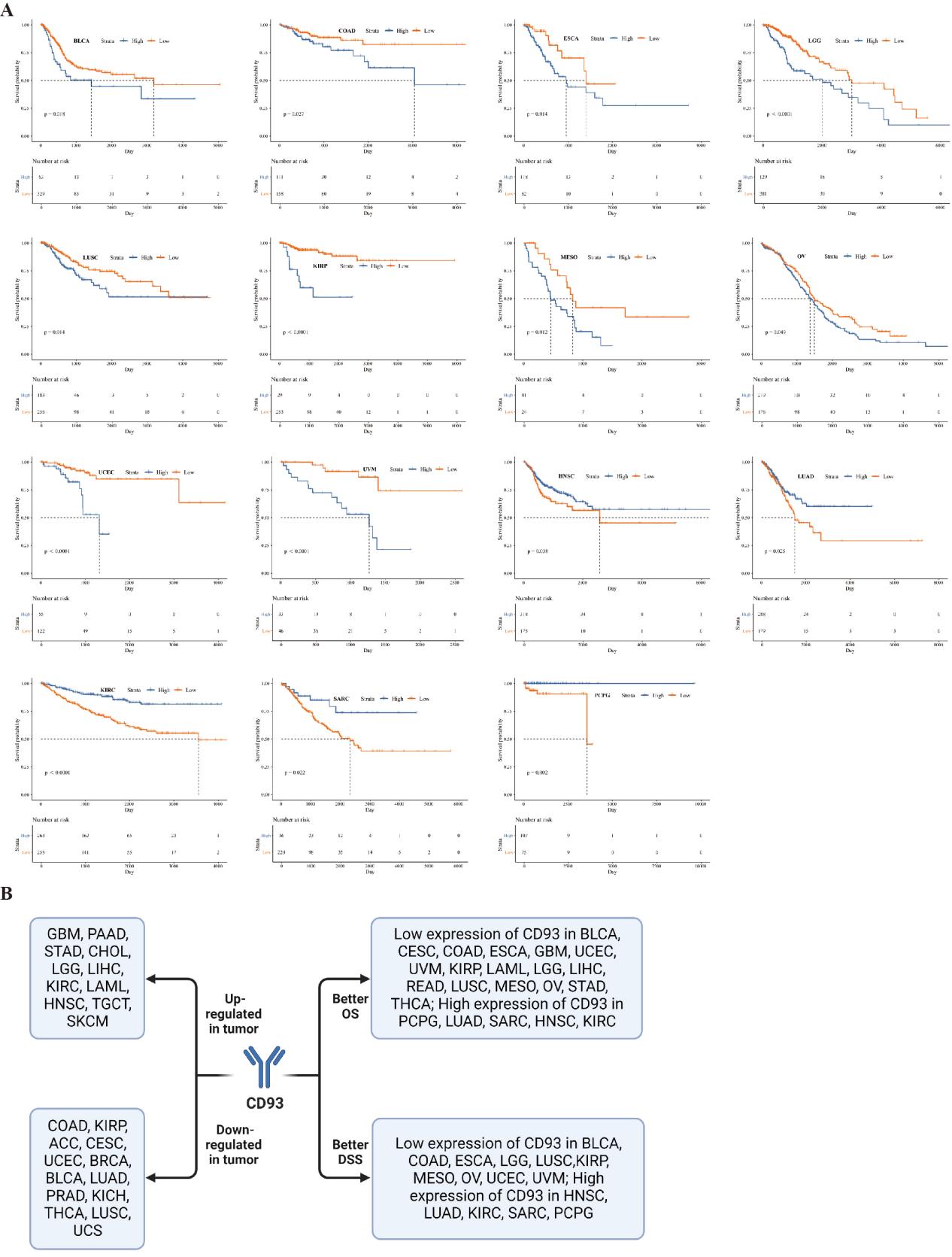
**

**Figure S2. The prognostic value of CD93 on DSS displayed by the KM analysis (A) and the summary of CD93 expression in pan-cancer and its relationship with prognosis (B).**

**.**

**
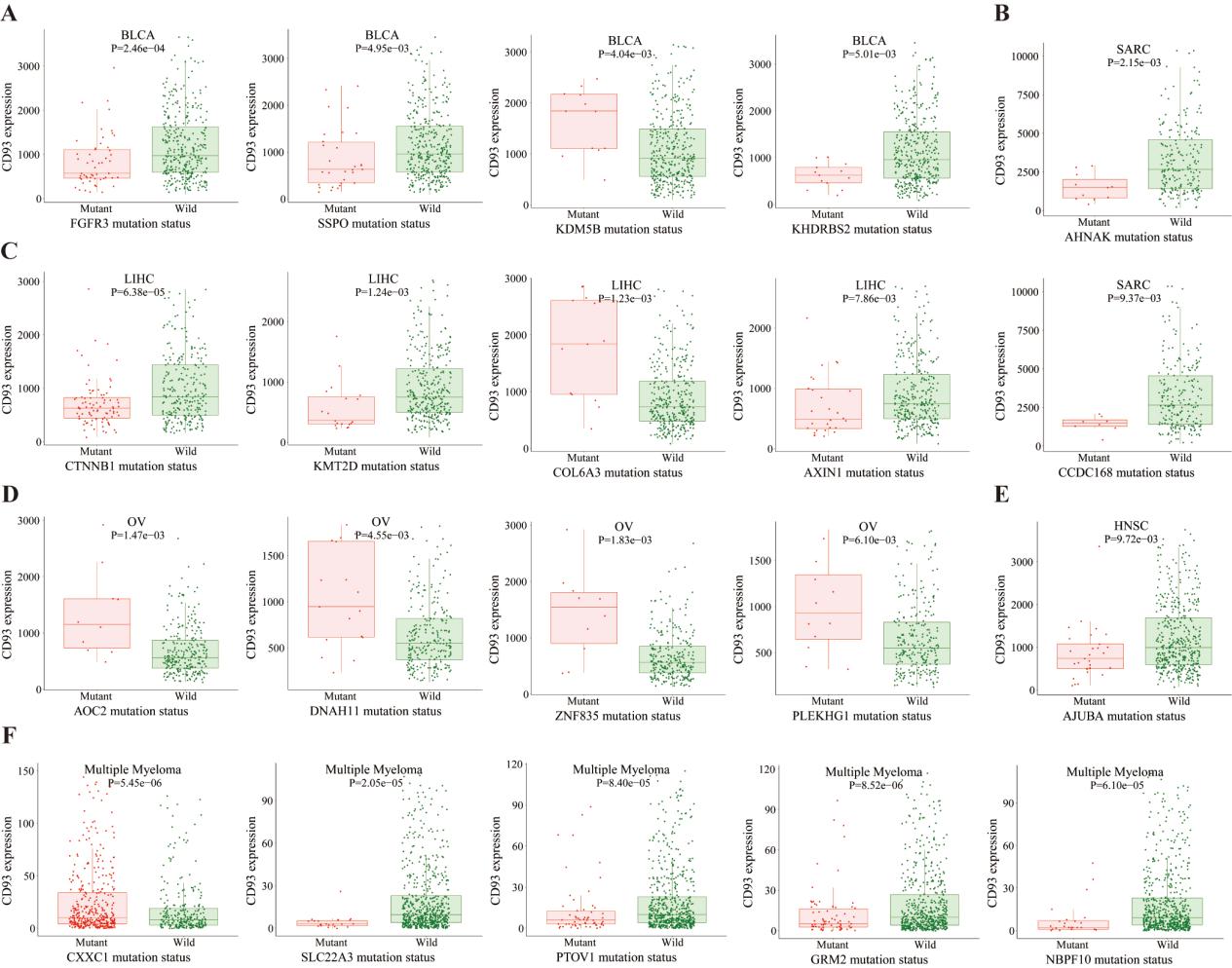
Figure S3. The correlation between CD93 expression and gene mutation status in pan-cancer were analyzed. BLCA (A), SARC (B), LIHC (C), OV (D), HNSC (E), and multiple myeloma (F).**

**
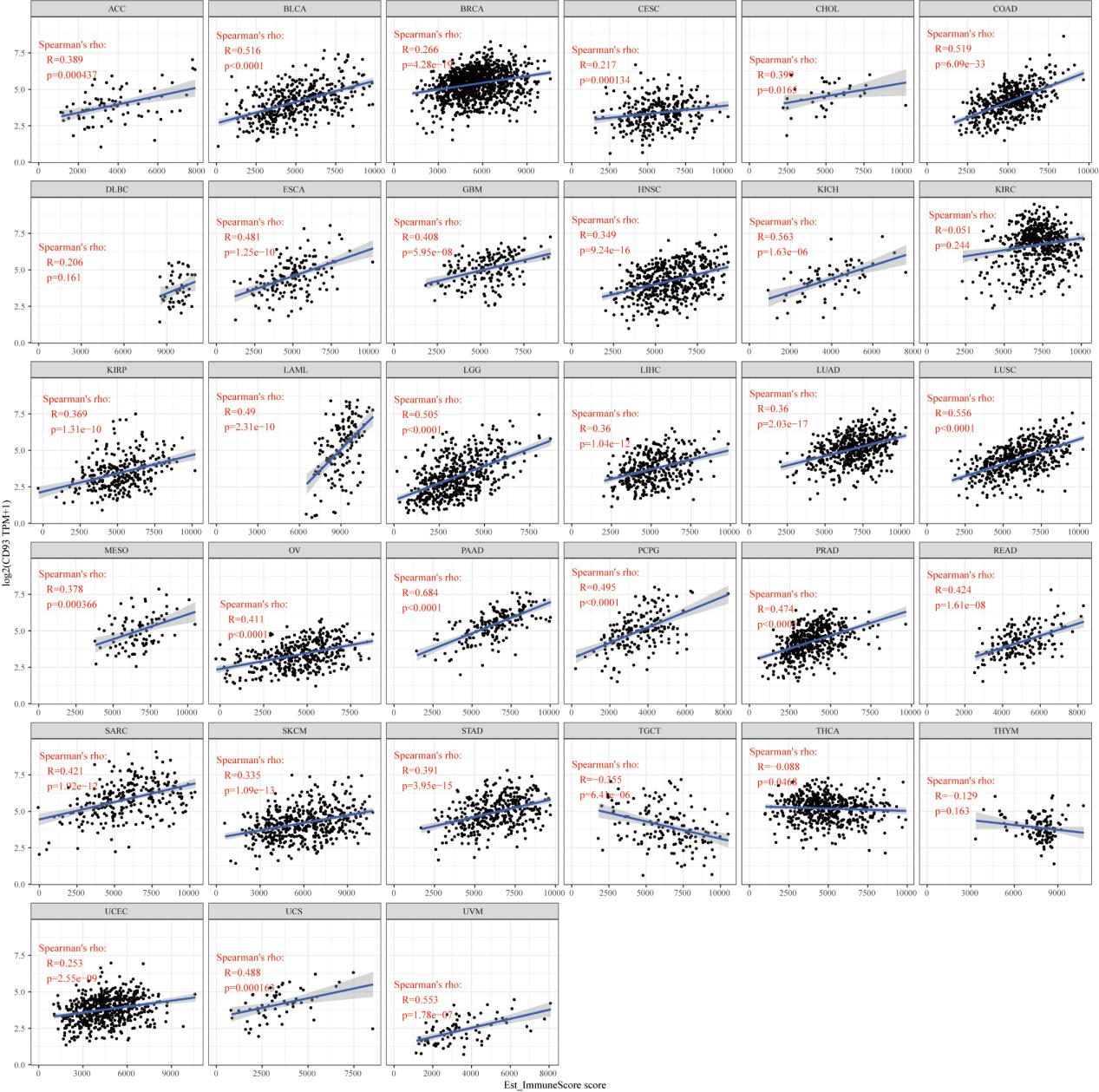
**

**Figure S4. Relationship between CD93 expression and the immune scores in pan-cancer.**

**
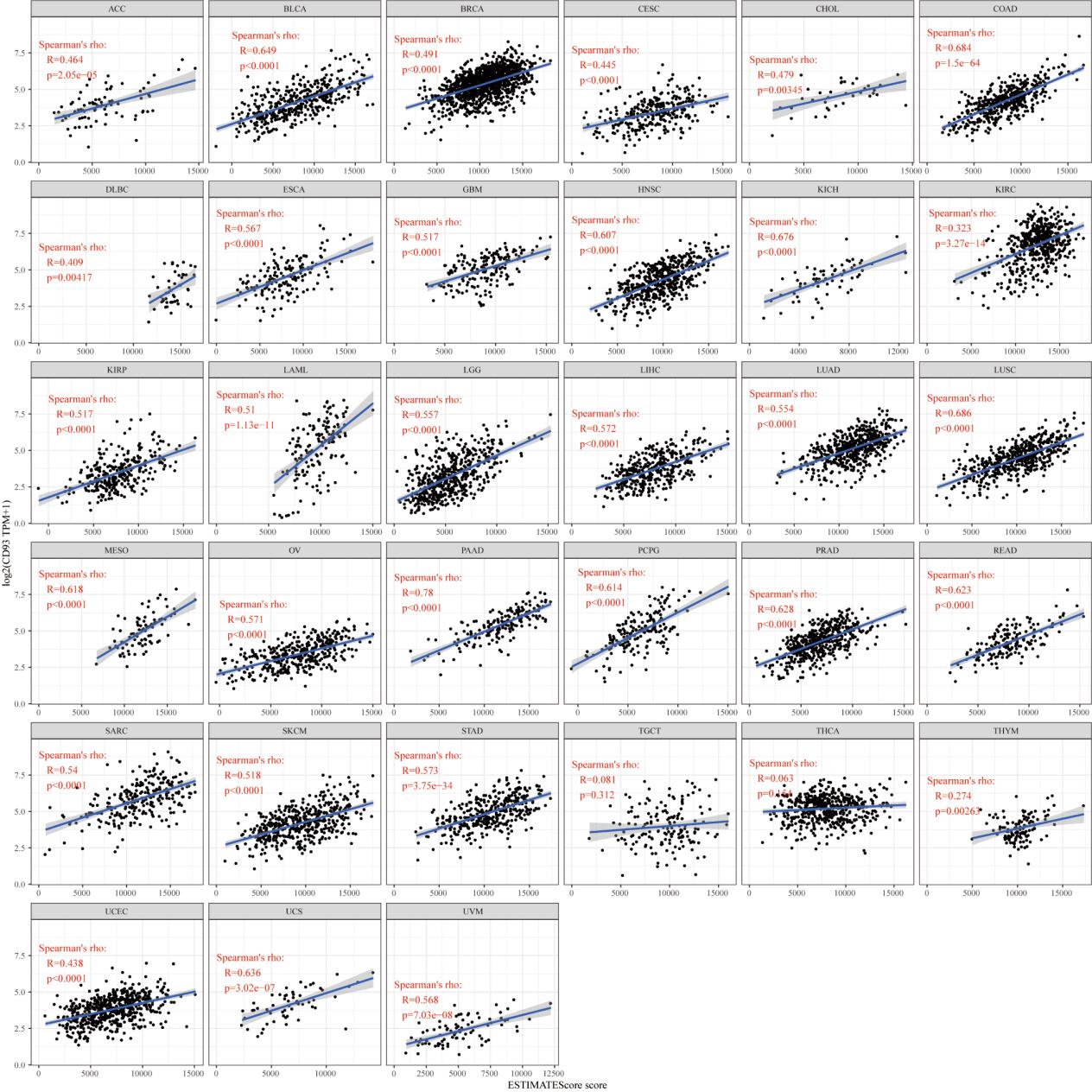
**

**Figure S5. Relationship between CD93 expression and the estimate scores in pan-cancer.**

**
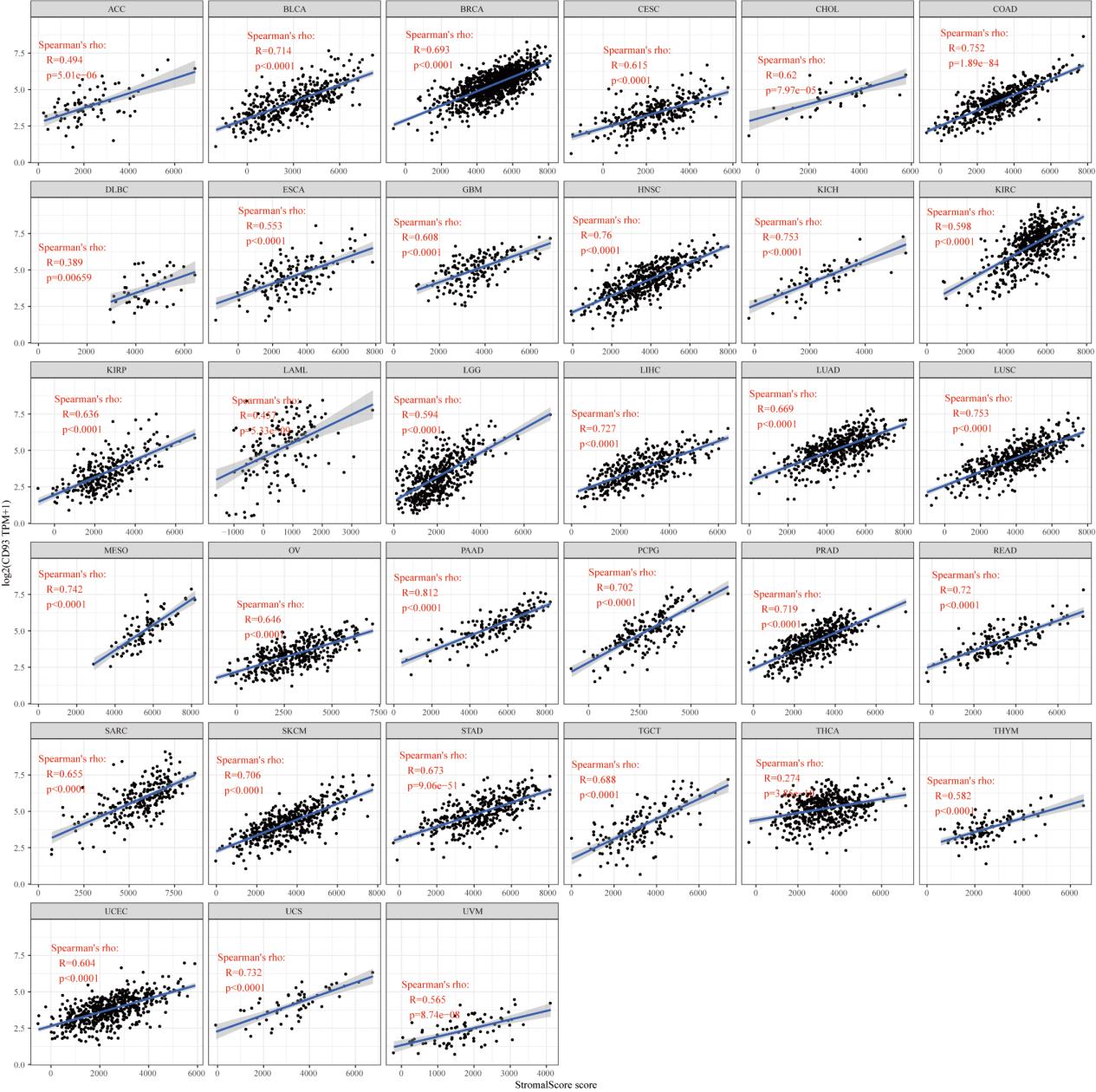
**

**Figure S6. Relationship between CD93 expression and the stromal scores in pan-cancer.**

**
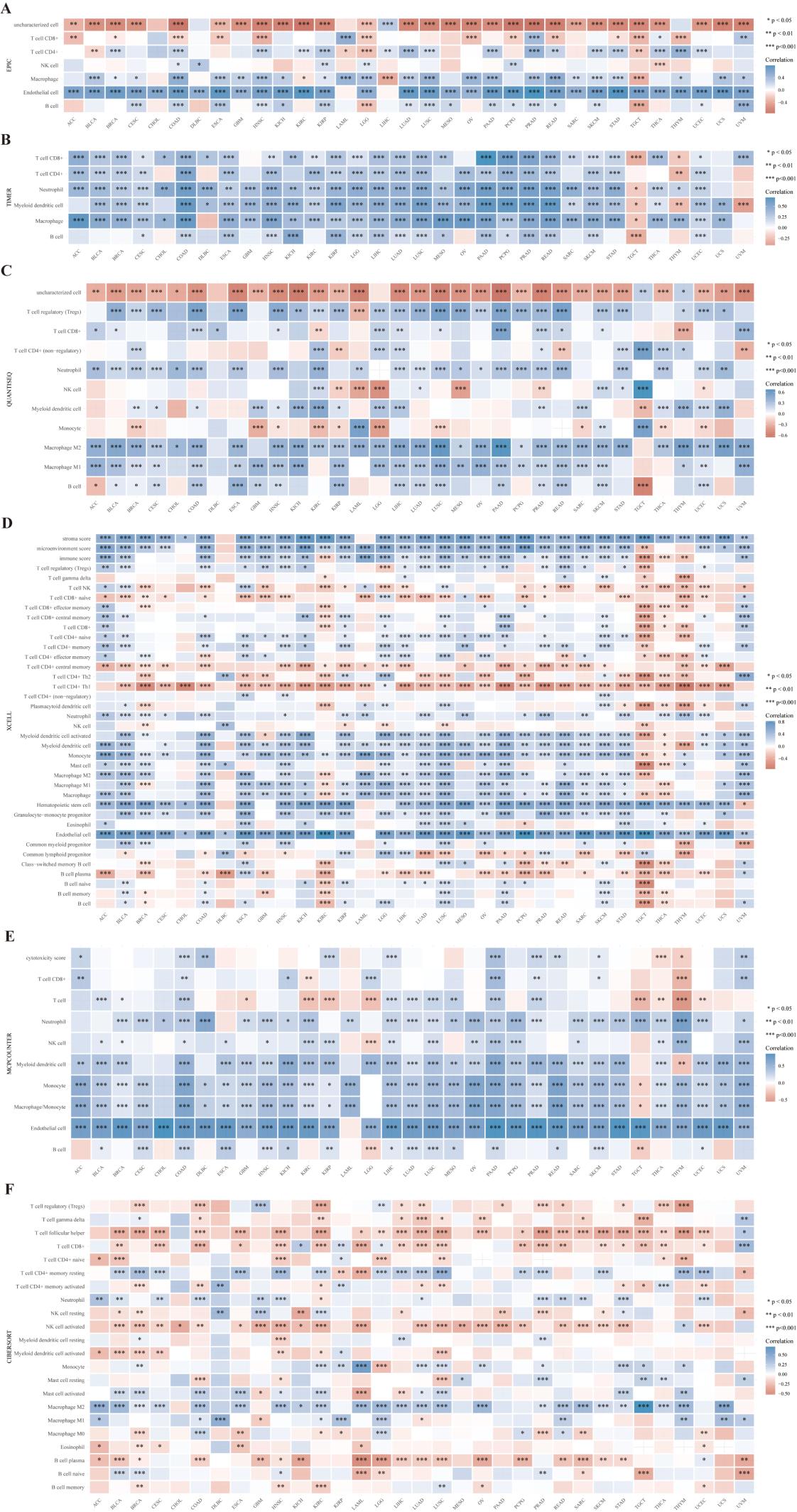
**

**Figure S7. Correlation between CD93 expression and immune infiltration in the TME. Immune cell infiltration identified by the EPIC (A), TIMER (B), QUANTISEQ (C), XCELL (D) , MCPCOUNTER (E), and CIBERSORT (F) algorithms. *p< 0.05, **p < 0.01, ***p < 0.001.**

**
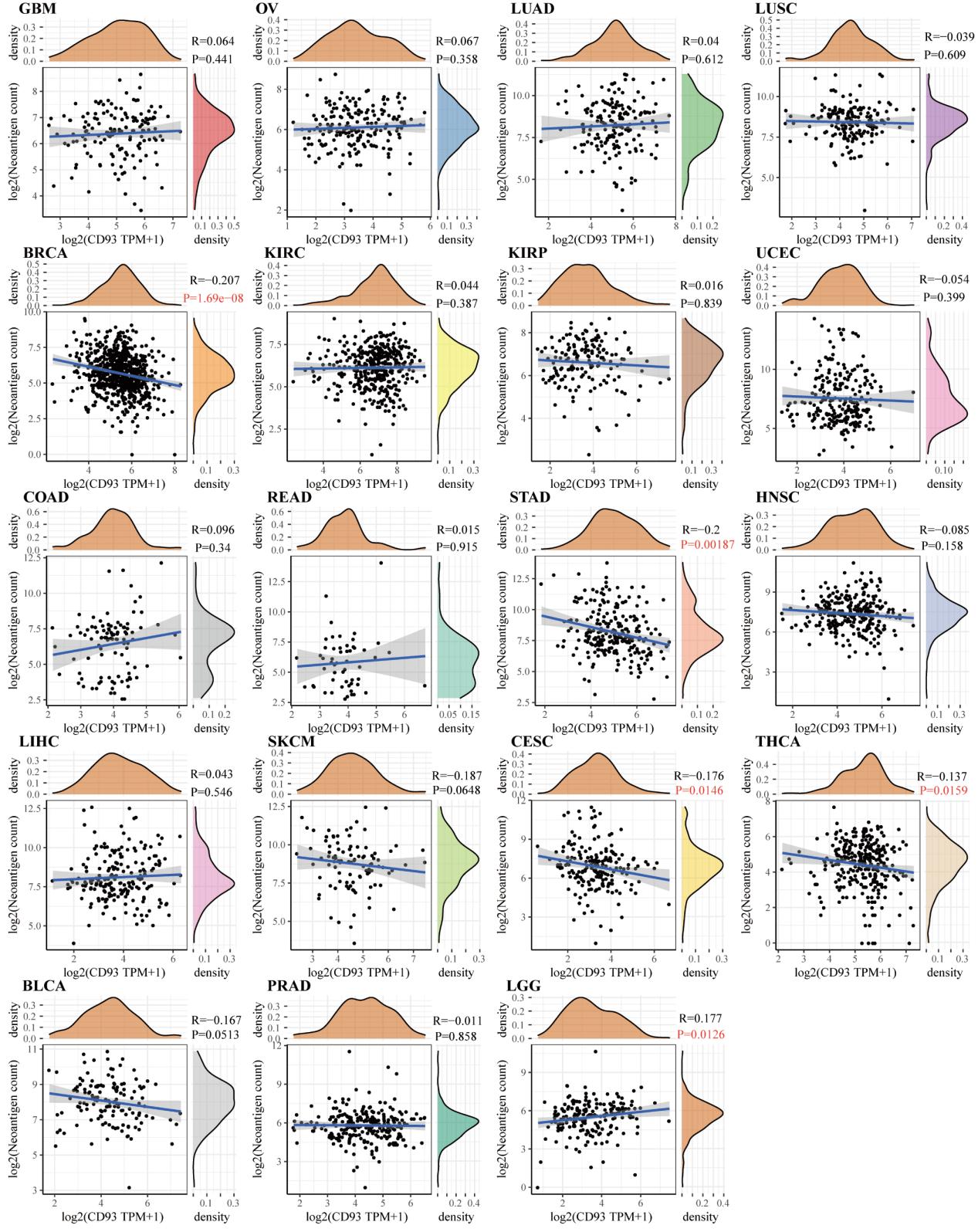
**

**Figure S8. Relationship between the expression of CD93 and neoantigens in pan-cancer.**

**
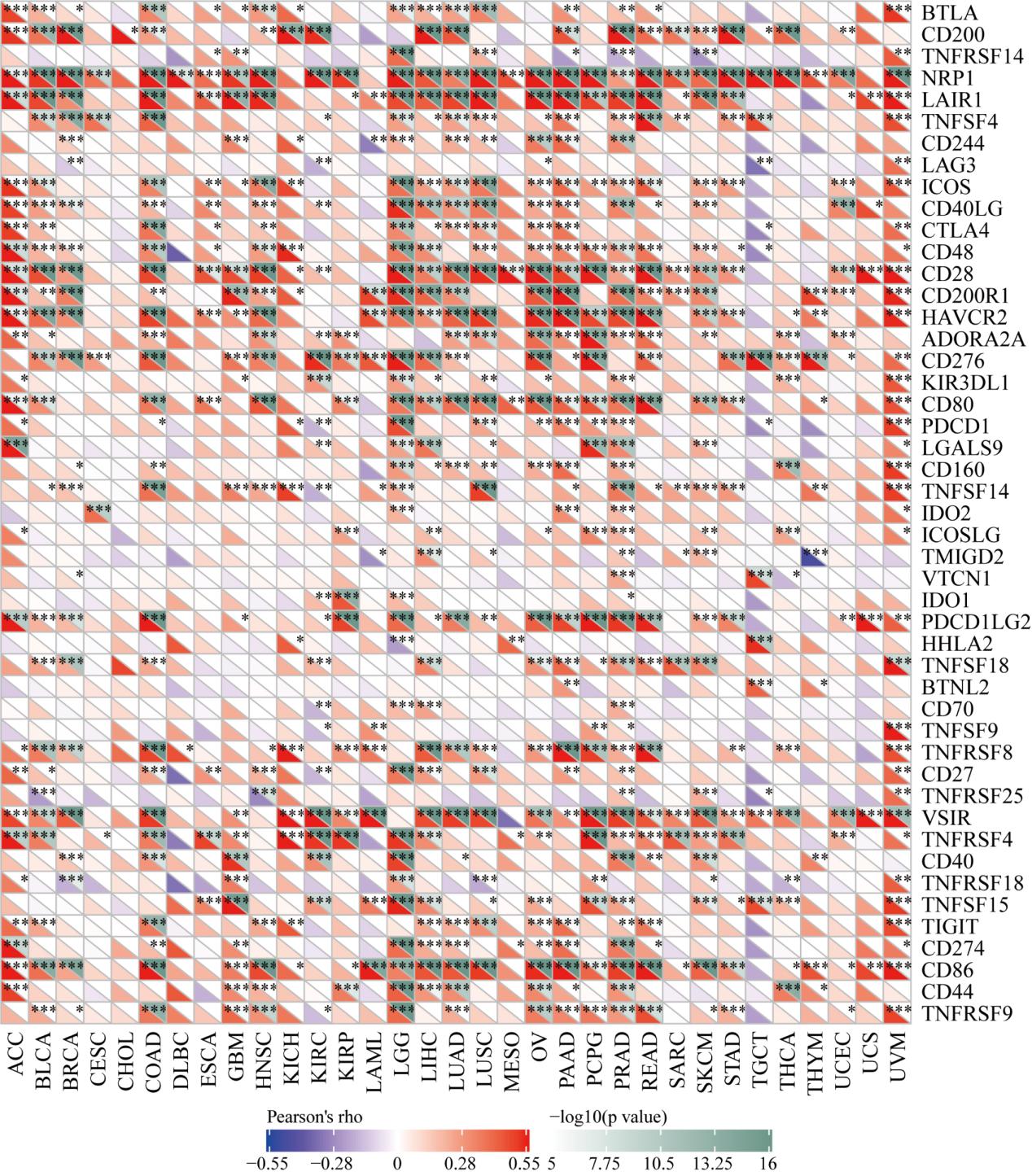
**

**Figure S9. Relationship between the CD93 levels and the expression of immune checkpoints in pan-cancer**

**
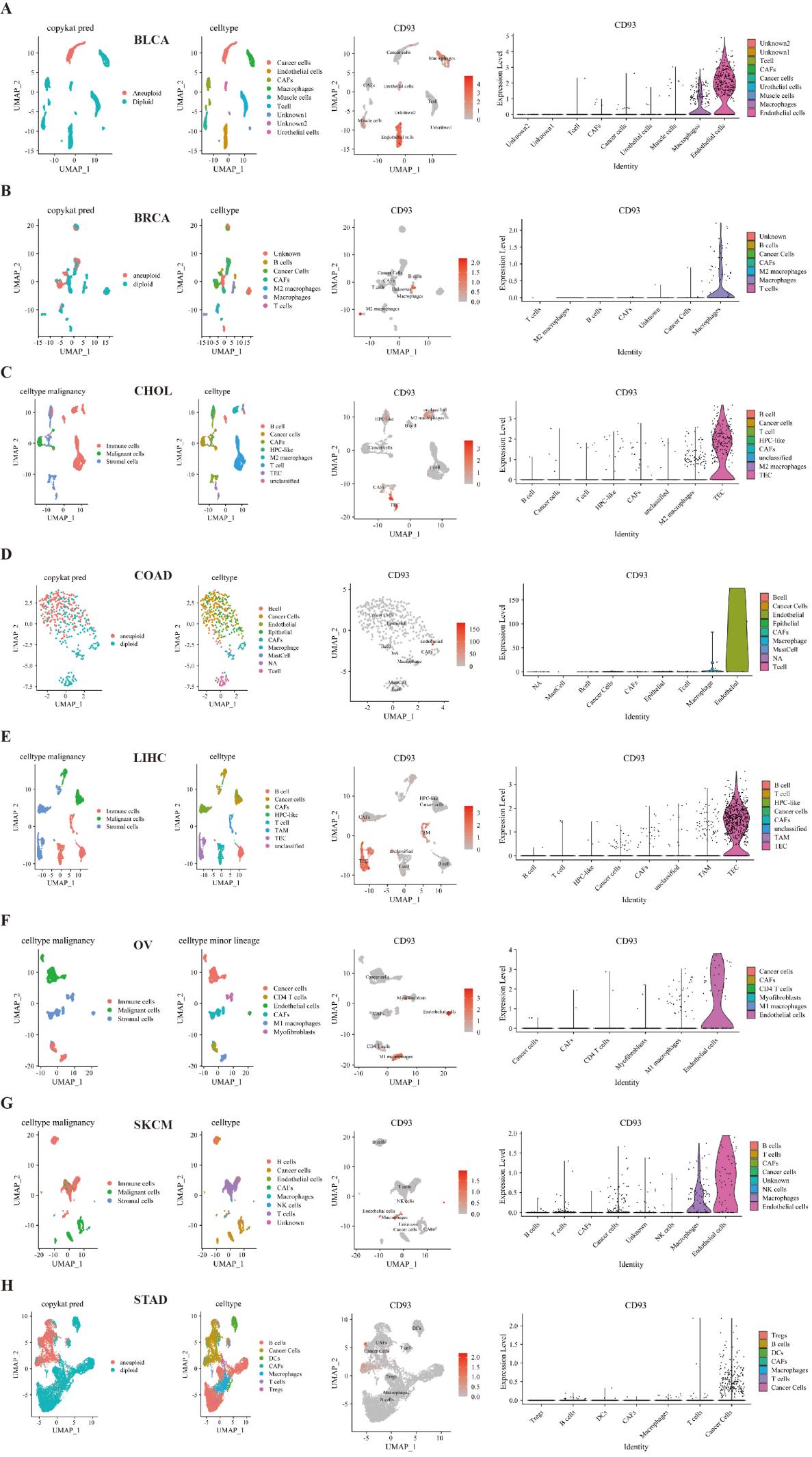
**

**Figure S10. Single cell sequencing analyzing the expression of CD93 on tumor and stromal cells in BLCA (A), BRCA (B), CHOL (C), COAD (D), LIHC (E), OV (F), SKCM (G), and STAD (H).**

**
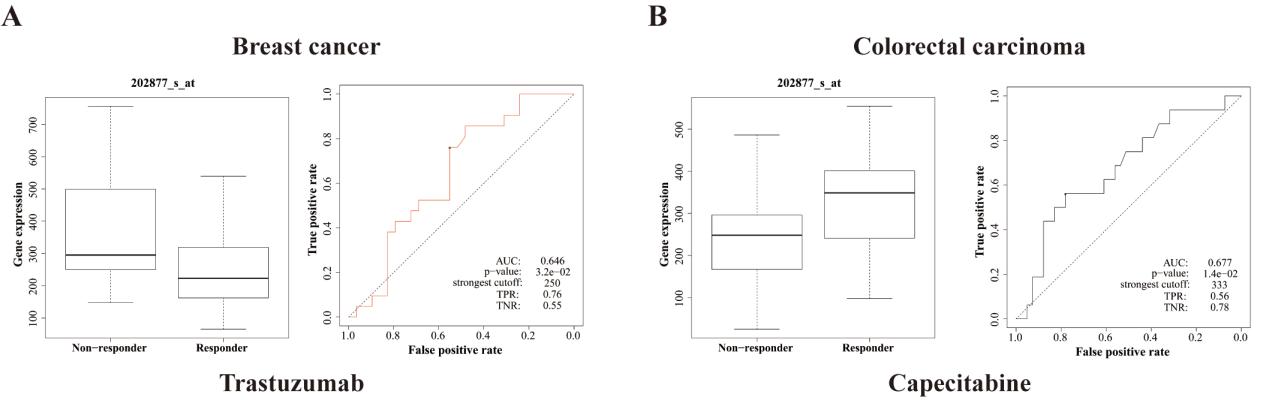
**

**Figure S11. The prediction of response to therapy targeting CD93 in breast cancer (A) and colorectal carcinoma (B) were analyzed from the ROC Plotter dataset.**

**Supplementary tables**

**Table S1. Survival difference between mutant and WT.**

**Table S2. Survival difference between CNV groups.**

**Table S3. The correlation between CD93 mRNA expression and CD93 methylation in pan-cancer.**

**Table S4. Survival difference between high and low methylation in each cancer.**

**Table S5. The correlation between CD93 expression and the sensitivity of CTRP and GDSC drugs in pan-cancer**

**Table S6. The correlation between CD93 expression and the sensitivity of CCLE and CellMiner compounds in pan-cancer.**
